# Comparative Efficacy of Metformin and Glimepiride in Modulating Pharmacological Network to Increase BDNF Levels and Benefit Type 2 Diabetes-Related Cognitive Impairment

**DOI:** 10.3390/biomedicines11112939

**Published:** 2023-10-31

**Authors:** Athira Anirudhan, Sheikh F. Ahmad, Talha Bin Emran, Paola Isabel Angulo-Bejarano, Ashutosh Sharma, Shiek S. S. J. Ahmed

**Affiliations:** 1 Central Research Laboratory, Believers Church Medical College Hospital, Kuttapuzha, Thiruvalla 689101, Kerala, India; 2 Department of Pharmacology and Toxicology, College of Pharmacy, King Saud University, Riyadh 11451, Saudi Arabia; 3 Department of Pathology and Laboratory Medicine, Warren Alpert Medical School, Brown University, Providence, RI 02912, USA; 4 Legorreta Cancer Center, Brown University, Providence, RI 02912, USA; 5 Department of Pharmacy, Faculty of Allied Health Sciences, Daffodil International University, Dhaka 1207, Bangladesh; 6 Regional Department of Bioengineering, NatProLab-Plant Innovation Lab, Tecnologico de Monterrey, Queretaro 76130, Mexico; 7 Drug Discovery and Multi-Omics Laboratory, Faculty of Allied Health Sciences, Chettinad Academy of Research and Education, Chettinad Hospital and Research Institute, Kelambakkam 603103, Tamil Nadu, India

**Keywords:** type 2 diabetes, brain-derived neurotrophic factor, metformin, glimepiride, protein network, gene and protein expression

## Abstract

Cognitive impairment is anotable complication of type 2 diabetes (T2DM), accompanied by reduced brain-derived neurotrophic factor (BDNF) in the brain and blood. Anti-diabetic drugs reduce hyperglycemia, yet their effect on cognitive improvement is unknown. We aimed to investigate the effect of anti-diabetic drugs regulating BDNF in T2DM through computational and case-control study design. We obtained T2DMproteins viatext-mining to construct a T2DMprotein network. From the T2DMnetwork, the metformin and glimepiride interactomes and their crucial shortest-path-stimulating BDNF were identified. Using qRTPCR, the genes encoding the shortest-path proteins were assessed in four groups (untreated-T2DM, metformin-treated, glimepiride-treated, and healthy controls). Finally, ELISA was used to assess serum BDNF levels to validate drug efficacy. As a result of this investigation, aT2DMnetwork was constructed with 3683 text-mined proteins. Then, the T2DMnetwork was explored to generate a metformin and glimepiride interactome that establishes the critical shortest-path for BDNF stimulation. Metformin stimulates BDNF via APP binding to the PRKAB1 receptor. Whereas, glimepiride increases BDNF by binding to KCNJ11 via AP2M1 and ESR1 proteins. Both drug shortest-path encoding genes differed significantly between the groups. Unlike metformin, BDNF gene and protein expression rise significantly with glimepiride. Overall, glimepiride can effectively increase BDNF, which could benefit T2DM patients with cognitive deterioration.

## 1. Introduction

Type 2 diabetes mellitus (T2DM) is a complex metabolic disorder, affecting millions of individuals worldwide [1]. T2DM is mainly characterized by hyperglycemia with abnormal secretion and actions of insulin. Genetic and environmental factors are the major causes for the development of T2DM [2]. Recent reports suggest insulin resistance leads to dysfunction in the central nervous system (CNS) that causes impairment in cognitive behaviors [3,4]. Such cognitive dysfunction causes a risk of dementia that affects quality of life. Current recommended anti-diabetic drugs are efficient and mainly targeted to increase insulin sensitivity and/or to stimulate beta cells to produce insulin. Of several anti-diabetic drugs, biguanide (metformin) and sulfonylurea (glimepiride) are used as first line oral drugs for T2DM patients [5,6]. The action of metformin helps to improve insulin sensitivity, whereas glimepiride stimulates pancreatic beta cells to produce sufficient insulin [7,8,9]. Both drugs reduce hyperglycemia, but their effect on cognitive improvement is unknown.

The brain-derived neurotrophic factor (BDNF) is a neurotrophin involved in neuronal maturation, repair, and synaptic plasticity in the central nervous system. BDNF is expressed at a higher level in the adult brain in order to maintain excitatory and inhibitory synaptic transmission and activity-dependent plasticity by regulating neuronal differentiation and survival. BDNF regulates the hippocampal long-term potentiation and long-term enhancement of synaptic efficacy implicated in learning and memory processes [10]. In addition to its effect on the nervous system, BDNF has also been linked to T2DM and other inflammatory disorders [11,12]. Several possible mechanisms were observed to link BDNF and T2DM. Teillon et al. conducted an experiment on animals in which decreased levels of PPAR-alpha and fibroblast growth factor led to BDNF-dependent insulin resistance and dyslipidemia [13]. Similarly, Yamanaka et al. report that BDNF resists the age-dependent rise in blood glucose that leads to the development of pre-diabetes in rodents [14]. Li et al. found a negative correlation between decreased serum BDNF levels, body mass index, and homeostatic model insulin resistance assessment [15]. Moreover, an inverse relationship was observed between serum BDNF concentration, glucose at fasting, and illness duration. Passaro et al. investigated the relationship between BDNF, T2DM, and dementia and confirmed the crucial role of BDNF in T2DM patients with dementia [16]. Consequently, BDNF has garnered considerable interest regarding its potential function in preventing the progression of T2DM [10,11]. Also, studies suggest that BDNF may be a future target for developing new anti-diabetic therapies.

**Figure 1:**
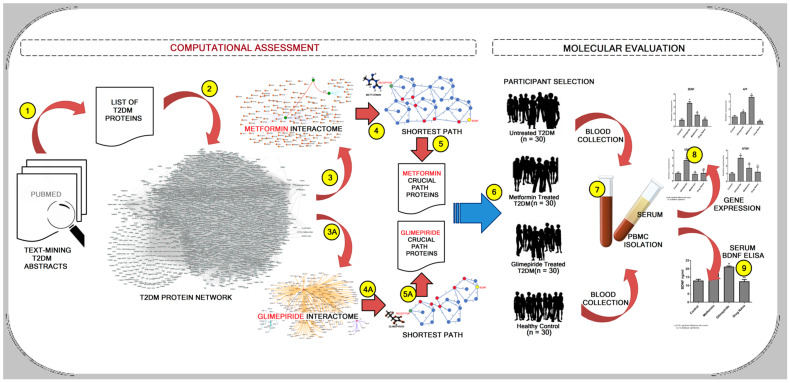
Computational and molecular approaches are part of the typical methodology for this investigation that adopts a computational and case-control study design. The computational procedures (Steps 1 to 5) include Step 1: text-mining selected abstracts from PubMed to compile a list of proteins associated with T2DM. Step 2: text-mined T2DM proteins are used to construct the T2DM-protein interaction network. Step 3: extraction of the metformin interactome from the T2DM-protein network, which provides all possible connections between the drug target receptors and BDNF, as well as the intermediating proteins. Step 4: the proteins involved in the shortest path connectivity between BDNF and the metformin target receptor were identified, and Step 5: the participating proteins were listed. Similar procedures were taken for glimepiride (Steps 3A–5A). Then, the molecular evaluation to computational outcome (Steps 6–9) was carried out. Step 6: case-control study design. All participants were recruited and categorized as follows: Group 1, newly diagnosed drug-naive T2DM patients (*n* = 30); Group 2, T2DM patients on metformin (*n* = 30); Group 3, T2DM patients on glimepiride (*n* = 30); and Group 4, healthy individuals (*n* = 30). Step 7 involved the collection of blood, followed by the isolation of peripheral blood mononuclear cells (PBMC) for Step 8: gene expression analysis. In Step 9, serum BDNF was measured using ELISA between the groups.

Despite the importance of BDNF in T2DM-dementia, the regulation and effect of current anti-diabetic medications on BDNF levels are not well understood and need to be clarified in detail at molecular levels. Using integrated computational and molecular approaches (Figure 1), the present study aims to investigate the behavior of metformin/glimepiride in activating BDNF through the signal transduction process in T2DM. Here we demonstrate a potential anti-diabetic drug that increases BDNF levels in terms of gene expression and serum protein levels, which may help to comprehend better treatment regimens for T2DM patients with cognitive impairment.

## 2. Materials and Methods

Our current methodology follows the strategic computational prediction of the crucial protein involved in BDNF stimulation and the subsequent use of a case-control study design for the validation of the crucial protein in T2DM patients and comparison with untreated and healthy controls.

### 2.1. Text Mining of T2DM-Associated Proteins/Genes

The NCBI, PubMed database was used to collect the abstracts using keywords related to T2DM, such as “Type 2 Diabetic Mellitus”, “T2DM”, “hyperglycemia”, and “Diabetes” in conjunction with “Homo sapiens” or “human” that were published between 1990 and April 2023. Two authors independently accessed the collected abstracts for their association with T2DM. Notably, these abstracts were then curated to check the studies presenting to humans subjects with T2DM and co-presenting gene and/or protein information. The articles presenting non-human studies, cell culture in vivo, meta-analysis, reviews, clinical reports, and case studies were eliminated before being subjected to the text-mining process [17,18]. Then, all curated abstracts were subjected to a text-mining procedure to retrieve the T2DM-reported proteins/genes using PubMed.mineR package [19]. Next, the point-wise mutual information method (polmineR) was implemented to weigh the protein/gene association with T2DM in each selected abstract [20]. Further, the collected gene/proteins were curated to remove duplicates for subsequent analysis.

### 2.2. Drug Interactomes and Functional Enrichment

From the text-mined proteins, the T2DM protein interaction network was constructed using the Cytoscapeplug-in. Simultaneously, metformin and glimepiride binding drug target receptors were collected from the Drug Bank Database (www.drugbank.ca, accessed on 16 August 2023) [21]. From the constructed T2DM protein network, two individual drug interactomes for metformin and glimepiride were extracted using NAViGaTOR (http://ophid.utoronto.ca/navigator/, accessed on 2 September 2023) [22], which provided all the possible connections between the drug target receptors leading to the BDNF, along with the intermediatory proteins. Then, the proteins in each drug interactome were subjected to gene ontology analysis using the EnrichR database [23]. Likewise, the molecular pathways associated with both the drug interactomes were determined by the KEGG pathway database [24]. The significance of these proteins relating to gene ontology as well as for molecular pathways was filtered by cut-off *p*-value < 0.01.

### 2.3. Critical Signals Transduction Process

Both sets of drug interactomes were mined to collect all conceivable signal cascades leading from drug receptors to BDNF, along with the intermediary proteins. Among these, the cascade with the shortest path connectivity between BDNF and the drug target receptors was identified by using the Cytoscape path analyzer plug-in [25]. This extracted shortest drug path identifies the necessary and immediate downstream signals that are activated by the drug (whether it be metformin or glimepiride), which connects BDNF proteins. In addition, the intermediary protein encoding genes of the shortest path, along with the BDNF, were evaluated for their gene expression in the PBMC of the participants who were under metformin or glimepiride treatment, compared with untreated T2DM patients and healthy controls, for further confirmation.

### 2.4. Recruitment of the Study Individuals

All participants involved in this study were recruited at Chettinad Hospital and Research institute, Kelambakkam, Tamil Nadu, India. This study was conducted according to the principles of the Declaration of Helsinki and appropriate ethical approval was obtained from the Institutional Ethics Committee of the Chettinad Academy of Research and Education (IHEC/1-19/Proposal No. 288) for the collection of peripheral blood from the participants. All participants were selected based on the following inclusion and exclusion criteria. Inclusion criteria were: (1) onset age of disease between 32–50 years, (2) no evidence of cognitive complaints in the individual, (3) no clinical evidence of diabetic complication, (4) absence of major repercussions in the activities of daily living, and (5) absence of dementia (based on the Diagnostic and Statistical Manual of Mental Disorders IV criteria). Among the selection criteria for the T2DM patients under metformin/glimepiride for a period of 15 to 18 months, controlled glycemic levels based on HBA1c and other clinical characteristics were recorded for all the participants of this study (Table 1). Alternatively, the exclusion criteria included HIV positive patients, those unwilling to give consent, patients with Type 1 diabetes, patients who were physically challenged, pregnant women and patients with diabetic complications, and patients under anti-drugs (other than metformin/glimepiride) and insulin therapy. Written informed consent was obtained from all study participants and they were classified into Group 1: newly diagnosed T2DM individuals-untreated (*n* = 30); Group 2: T2DM individuals under metformin medication (*n* = 30); Group 3: T2DM individuals under glimepiride medication (*n* = 30); and Group 4: healthy individuals (*n* = 30).

### 2.5. RNA Isolation from Peripheral Blood Mononuclear Cells

First,5 mL fresh EDTA-blood samples were obtained from the participants. Then, the peripheral blood mononuclear cells (PBMCs) were isolated using Histopaque-1077 (Sigma–Aldrich, Burlington, MA, USA), according to standard protocol, by overlaying the blood on a density gradient solution and with centrifugation at 1500–1800 rpm for 30 min. The buffy coat layer containing the PBMCs was aspirated, washed thrice with phosphate-buffered saline, and PBMC pellets were used for RNA isolation [26]. Total RNA was isolated using the TRIzol™ LS Reagent (Thermo Fisher Scientific, Waltham, MA, USA) [27] according to the protocol provided by the manufacturer. Using a NanoDrop spectrophotometer 2000 (Thermo Fisher Scientific, Waltham, MA, USA), total RNA concentrations were measured spectrophotometrically at 260/280 nm [28]. Electrophoresis on a 1% agarose gel and a Bioanalyzer 2100 (Agilent, Santa Clara, CA, USA) were used to assess the quality of the isolated RNA and its integrity, respectively.

### 2.6. cDNA Synthesis and Gene Expression Analysis

cDNA was synthesized using a Reverse Transcriptase Core Kit (Eurogentec, Seraing, Belgium) with 100 ng of total RNA introduced to the reaction containing reverse transcriptase, 10 µM oligo dT, 10 mM dNTPs, and 40 units of RNA inhibitors. The reverse transcriptase reaction was performed in an Applied Biosystems PCR System 9902 by incubating the reaction mixture at 42 °C for 60 min for first strand synthesis, followed by 10 min at 95 °C to deactivate the enzyme. The synthesized cDNA was kept at −20 °C until further processing. The quantitative real-time PCR (qPCR) reaction was performed in 20 µL final volume using TB Green Advantage qPCR Premix (Takara Bio, San Jose, CA, USA,), nuclease-free water, a cDNA template, and appropriate primers (primers specific to the genes such as APP, GAPDH, AP2M1, ESR1, and BDNF). Primers were obtained from Sigma–Aldrich in a dry form and dissolved in nuclease-free water, according to the recommendation, to make 100 µM solutions. The PCR amplification was carried out using an Applied Biosystems PCR system ABI-7000 with cycle conditions (Initial Denaturation 95 °C 10 s, Denaturation 95 °C 5 s and Annealing/Extension 60 °C 32 s). All analyzed gene expressions were normalized using the GAPDH housekeeping gene. Then the relative gene expression was calculated by the 2^−ΔΔCT^ method. The primer sequences of specific genes in this study are listed in Table 2.

### 2.7. Estimation of Serum BDNF Levels

BDNF was detected in sandwich enzyme-linked immunoassay (Quantikine^®^ ELISA Human BDNF Immunoassay; R&D Systems, Minneapolis, MN, USA) according to the manufacturer’s protocol. The wells were prepared with blood serum samples and measured at 540/570 nm. The BDNF was quantified against a standard curve with known amounts of protein. Experiments were performed in triplicate, and the BDNF concentrations were expressed as ng/mL. The sensitivity ranges of BDNF were estimated based on standard curves. The significance of variation in BDNF levels between the groups was statistically assessed.

### 2.8. Statistical Analysis

All statistical analyses were performed utilizing SPSS software version 20.0. The clinical data, consisting of numerical values, were reported as the average ± standard deviation. In order to examine the discrepancy in gene expression and serum BDNF levels among the studied groups, a one-way analysis of variance (ANOVA) was conducted with Tukey’s HSD post-hoc test. Statistical significance was established using a threshold of *p* < 0.05. Additionally, the figures illustrating the gene expression and the serum BDNF protein between the groups were generated using GraphPad Prism software version 6.

## 3. Results

Herein, we implemented an integrated computational and molecular approach to determine the regulatory association of T2DM drugs in the improvement of BDNF levels to protect cognitive behavior in patients with T2DM.

### 3.1. Text-Mining and T2DM Network

**Figure 2:**
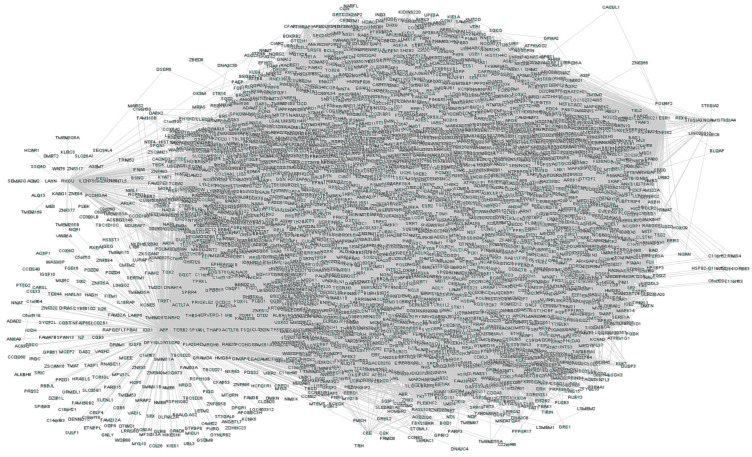
Network presenting the protein-protein interactions relevant to Type 2 diabetes. The proteins in the network were obtained through text-mined proteins of T2DM containing 3683 proteins with 12,521 connected edges. Nodes represent individual protein, while lines indicate the interactions.

**Figure 3:**
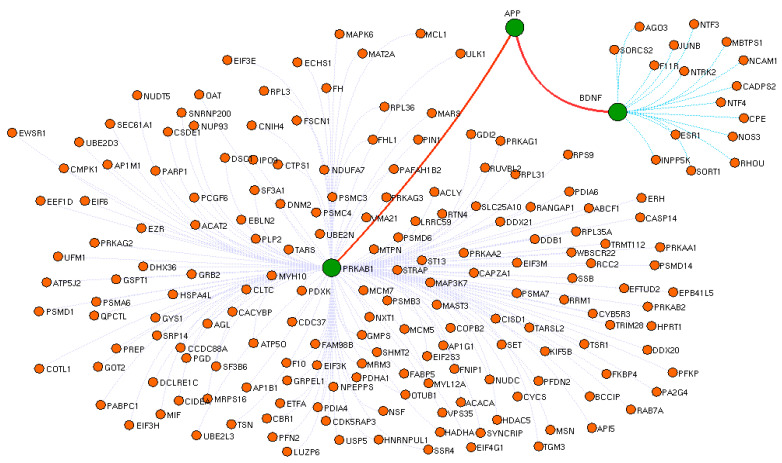
Molecular interactome of metformin in its possible connectivity in activating Brain-Derived Neurotrophic Factor (BDNF). Nodes (orange) represent proteins involved in this interactome, while gray lines represent its connectivity. The highlighted node (green) represents the shortest path traveled (edges: red) by a metformin receptor along with the intermediatory protein to activate BDNF. Blue dotted line represents the receptor node; purple dotted line represents the target node.

**Figure 4:**
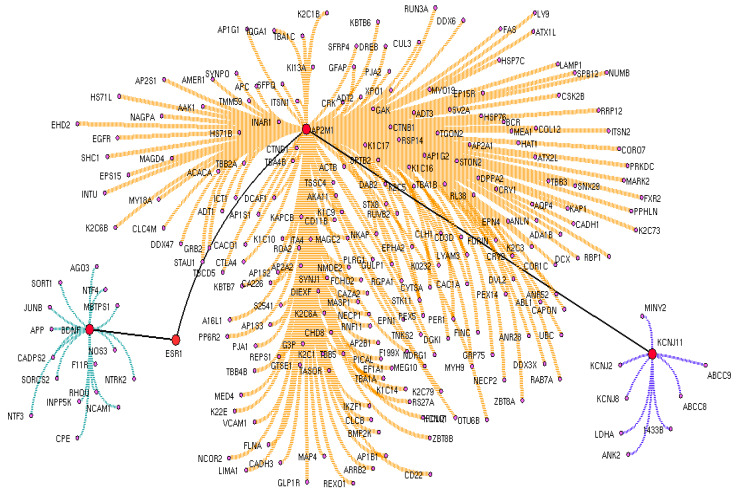
Molecular interactome associated with glimepiride signaling to activate Brain-Derived Neurotrophic Factor (BDNF). Each node in the diagram represents a protein (colored blue), the connectivity is represented as dotted lines(receptor closed connected proteins and BDNF closed connected proteins are represented with blue edges, the intermediary proteins with orange edges). The highlighted node (red) represents the shortest path traveled (edges: black) by the glimepiride receptor along with the intermediatory protein to activate BDNF.

Firstly, the list of proteins associated with T2DM were text-mined from the PubMed abstracts. Using 3683 proteins collected from the abstracts, a T2DM protein network was constructed having 12,521 connected edges (Figure 2). To the T2DM protein network, we extracted the drug interactomes of metformin and glimepiride, respectively. Both drug interactomes contain drug binding target receptors as an initial protein node that extends to reach BDNF with all possible intermediatory proteins. For instance, metformin is bound to the drug target receptor (PRKAB1) that acts as an initial node that connects with 191intermediate proteins that elongate to reach BDNF as the final node (Figure 3). Likewise, the glimepiride interactome was initiated via its drug target receptor (KCNJ11) that extended to BDNF with an intermediate 253 protein edges (Figure 4).

### 3.2. Drug Interactome Enrichment Analysis

Both the interactomes were enriched based on gene ontology (Figures S1 and S2) and molecular pathways (Table S1). The metformin network showed involvement in a variety of molecular functions such as AMP transport, phenylpropanoid catabolic process, octopamine metabolic process, and NAD catabolic process; whereas, the glimepiride was involved in the regulation of choline O-acetyltransferase activity, glyoxal metabolic process, dopamine biosynthetic process from tyrosine, and notochord cell differentiation function. Further, molecular functional enrichment shows the metformin interactome majorly participates in the JNK signaling pathway, S1P1 pathway, and ATM pathway. Likewise, the glimepiride interactome showed involvement in various Notch signaling pathways, Glypican pathways, and VEGFR1-specific signaling molecular pathways.

### 3.3. Crucial Protein in Drug Interactome

From both the drug interactomes, the critical downstream signal cascade for BDNF activation via shortest drug target path was identified using the Cytoscape plug-in. Further, the intermediatory protein encoding genes, along with the drug target receptors and the BDNF, were assessed for their gene expression in PBMC, including those who were untreated T2DM (Group 1), under metformin (Group 2), under glimepiride treatment (Group 3), and the healthy control (Group 4). From the drug interactomes, the shortest BDNF activated paths between the drug target receptors and BDNF were extracted (Figure 3 and Figure 4). For metformin, the identified shortest path initiated from PARKAB1 (Protein Kinase AMP-Activated Non-Catalytic Subunit Beta 1) receptors that directly connects BDNF via Amyloid Beta Precursor Protein (APP). Alternatively, glimepiride initially interacted with KCNJ11 as the primary drug target receptor, which activated BDNF via downstream activation of AP2M1 and ESR1.

### 3.4. Gene Expression Analysis

**Figure 5:**
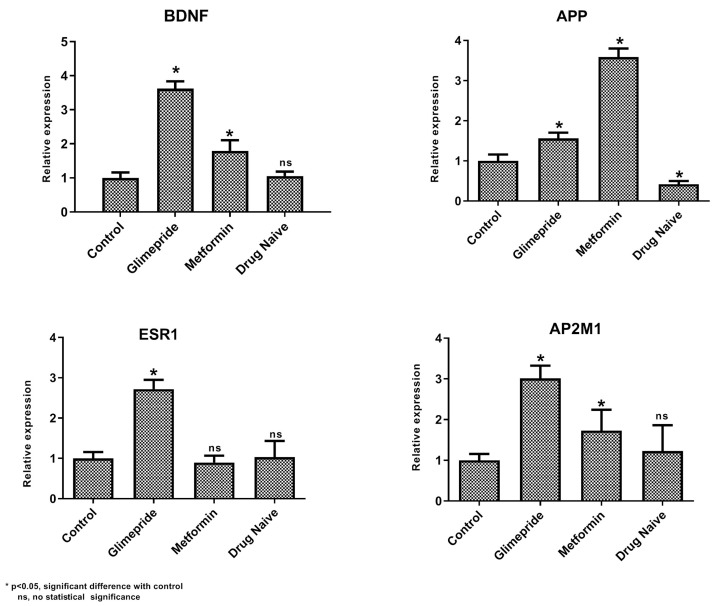
Difference in the expression level of BDNF, APP, ESR1, and AP2M1 genes among untreated T2DM (Group 1), metformin treatment (Group 2), glimepiride treatment (Group 3), and the healthy control (Group 4). Values are mean ± SD for the 30 samples in each group. * indicates the statistical significance (*p* < 0.05) difference in expression levels between the control and the respective groups; ns indicates insignificant.

**Figure 6:**
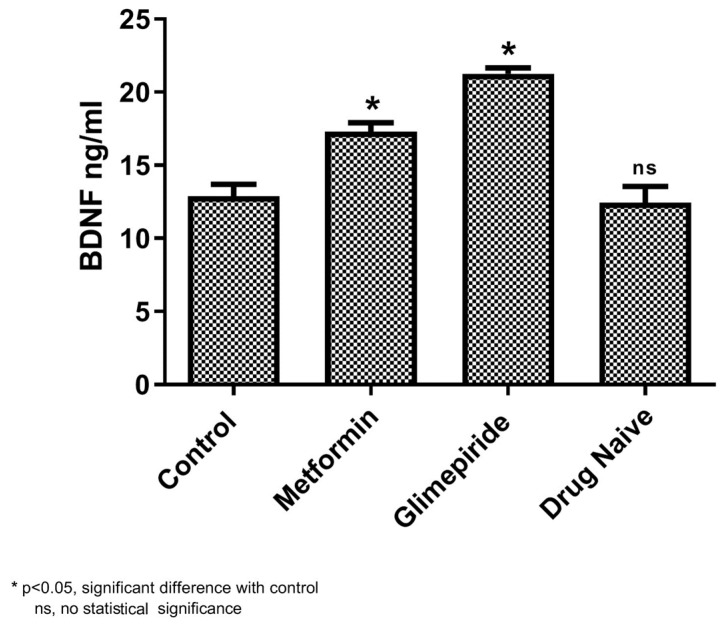
Serum BDNF protein concentration (ng/mL) in untreated T2DM (Group 1), under metformin (Group 2), under glimepiride treatment (Group 3), and in the healthy control (Group 4). Values are mean ± SD for the 30 samples in each group. * indicates the statistical significance (*p* < 0.05) difference in BDNF levels between the control and the respective groups; ns indicates insignificant.

The gene expression of BDNF and the intermediatory protein encoding genes for both the drugs were analyzed using qPCR. Analyzing the metformin shortest path network shows upregulation of APP and BDNF compared with untreated T2DM and the control (Figure 5). Similarly, in glimepiride, upregulation of BDNF along with AP2M1 and ESR1 were noticed compared to the untreated T2DM and control group (Figure 5). Interestingly, an increased BDNF level was noticed in the glimepiride group in comparison to the metformin group. Additionally, the increased serum BDNF observed in T2DM patients under metformin and glimepiride therapy also supports our gene expression results (Figure 6).

## 4. Discussion

Extensive studies have been undertaken to investigate the relationship between low BDNF levels and central nervous system disorders. Furthermore, meta-analyses have shown that BDNF levels change in neurological illnesses such as Parkinson’s disease and Alzheimer’s disease, as well as in psychiatric disorders such as autism, depression, post-traumatic stress disorder, and anxiety disorders [29,30,31,32,33,34]. Comparatively, only a few studies have been conducted to examine the levels of BDNF in individuals with T2DM. The majority of these investigations found a significant decrease in BDNF levels in T2DM patients compared to controls [35,36]. These findings show a strong relationship between T2DM and neurological and psychosocial complications. T2DM, in particular, has been linked to an increased risk of developing depression, anxiety disorders, and dementia [37,38]. According to Krabbe et al., decreased levels of BDNF are related to impaired glucose metabolism in persons with T2DM [39]. Furthermore, it has been discovered that advanced glycation end products play a crucial role in the development of T2DM-related disorders by producing oxidative stress, inflammation, and vascular damage [40]. BDNF has been shown to suppress the expression of advanced glycation end receptors and the associated nuclear factor kappa B (NF-κB) signaling pathway [41]. These findings imply that low levels of BDNF may have a major impact on the onset and progression of T2DM. Furthermore, studies suggest that BDNF regulates glucose-related metabolic processes. As a result, it is possible that T2DM could influence the regulation of BDNF levels in body fluids. Furthermore, given the evidence that BDNF is involved in the regulation of the central nervous system and metabolic processes, alterations in circulating BDNF levels may precede the onset of T2DM. However, the current understanding of how anti-diabetic medicines influence BDNF alterations in people with T2DM is unclear. We investigated the BDNF signal cascade using a computational approach, then performed molecular validation based on a case control study design, which revealed that metformin and glimepiride had a positive effect on BDNF gene expression and protein levels in serum when compared to untreated T2DM and healthy individuals.

Several investigations have demonstrated the modulation of BDNF levels by anti-diabetic medications in various experimental settings, including in vitro, animal, and human models [42,43,44,45,46]. Yoo et al. [43] demonstrated the correlation between BDNF levels and the administration of metformin, both as a standalone treatment and in combination with glimepiride, in a murine model. Glimepiride, classified as a second-generation sulfonylurea, exerts its effects by binding to specific sulfonylurea receptors on pancreatic beta cells. This binding leads to an increase in endogenous insulin production and the subsequent activation of intracellular insulin receptors. The intervention leads to a decrease in HbA1c and fasting glucose levels. Nevertheless, it is worth noting that weight gain and hypoglycemia are frequently observed as negative outcomes associated with the use of all sulfonylureas [47]. Metformin, classified as a biguanide anti-diabetic medication, exerts its effects by decreasing hepatic glucose production and inhibiting glucose absorption through the gastrointestinal tract. Additionally, it enhances insulin uptake in peripheral tissues, hence improving insulin sensitivity in both hepatic and peripheral tissues. The intervention results in reductions in HbA1c and fasting glucose concentrations, as well as decreases in plasma triglyceride and low-density lipoprotein (LDL) cholesterol levels. In the animal model, following a duration of 5 weeks on a high-fat diet, oral administration of metformin alone or in combination with glimepiride was conducted once daily for a period of 3 weeks. The administration of metformin, either as a standalone treatment or in combination with glimepiride, was found to be associated with a reduction in weight gain and food consumption. In addition, the group that received the high-fat diet together with the vehicle treatment exhibited a notable decrease in BDNF protein levels inside the dentate gyrus, an area responsible for receiving inputs into the hippocampus, in comparison to the group that received the low-fat diet treatment. In the high-fat diet group, the administration of metformin or metformin in combination with glimepiride effectively prevented a decrease in BDNF levels [43]. Similar results were observed in our study, confirming the efficacy of the drugs that improve BDNF gene expression and protein levels in T2DM. Interestingly, our computational assessment generates metformin and glimepiride interactomes which depict the multiple intermediatory connections between the drug target receptors and BDNF.

The enrichment analysis of drug interactomes reveals these genes were involved in various other cellular functions. Moreover, the molecular pathway of these two drugs showed activating 37 common pathways, including the EGF receptor (ErbB1) signaling pathway, the EphrinB-EPHB pathway, the EPO signaling pathway, the FGF signaling pathway, the insulin pathway, the mTOR signaling pathway, the p53 pathway, and the S1P1 pathway. However, we investigated the shortest path of these drug interactomes, containing their drug target receptors, intermediate proteins, and BDNF. In metformin, APP proteins connect directly between the metformin target receptor and BDNF. Studies report that amyloid precursor protein (APP) and amyloid-β-peptide (Aβ) are involved in the dysfunction of mitochondrial activity, which increases oxidative stress in mitochondria and impairs the key glucose metabolic pathway involved in disease pathogenesis [48,49,50]. Likewise, the glimepiride network had AP2M1 and ESR1 as intermediatory proteins that connect the drug target receptor and BDNF. Recent reports suggest impaired glycemic homeostasis was observed in men with estrogen resistance and mutation in ESR1 and CYP19A1 [51,52,53]. In the brain, the estrogen-dependent signaling is mediated through intracellular, transmembrane and membrane-bound estrogen receptors (ERs). The systemic review by Sundermann et al. revealed the strong association between ESR1 mutation and cognition [54]. Similarly, the transcriptomics profiling of beta cells in the pancreas reveals several genes that drive T2DM, in which AP2M1 is a potential player in the regulation in synaptogenesis signaling pathways. Interestingly, the intermediators of both drugs showed down regulation in untreated T2DM patients. Whereas these proteins were upregulated on metformin and glimepiride therapy, this clearly denotes the drug plays a crucial role in the maintenance of BDNF levels. Particularly in glimepiride therapy, AP2M1, ESR1, and BDNF were increased when compared to metformin treatment, which suggests glimepiride activates the AP2M1 and ESR1 that contribute to the increased levels of BDNF. Alternatively, APP was increased in metformin therapy, which also increases BDNF, but not like that of glimepiride. Thus, glimepiride is suggested as effective in increasing BDNF and benefits T2DM in improving cognitive decline. Overall, our study establishes the anti-diabetic drug-targeted interactomes and their interplay in T2DM regulation. This approach helps to identify the shortest path signal of the most frequently recommended anti-diabetic drugs, metformin and glimepiride, that activate BDNF, a key molecule involved in cognitive behavior. Our results revealed the utility of drugs for the effective treatment of T2DM patients with cognitive decline. Also, gene ontology and molecular pathway enrichment analysis supports the significance of these intermediatory proteins and their role in T2DM, which was further supported with gene and serum protein expression. Although our study benefits the clinician for the recommendation of the treatment, it has limitations with respect to the limited participants enrolled in each group. Hence, analyzing more samples would prove the significance of interactomes and alternate intermediate targets for drug development.

## 5. Conclusions

In conclusion, our investigation, which employed a computational and molecular approach, unveiled an encompassing function of anti-diabetic medications for stimulating BDNF in individuals with T2DM. By emulating computational methods, we identified distinct activation signaling cascades that facilitate the activation of BDNF via metformin and glimepiride. Further molecular validation through the utilization of qRTPCR and ELISA demonstrated that glimepiride is superior to metformin in its ability to increase BDNF levels. This differential effect may potentially benefit cognitive function in individuals with T2DM.

## Figures and Tables

**Table 1:** Clinical characteristics of all the participant grouped in this study.

Clinical Characteristics of Participants	Group 1: Untreated T2DM	Group 2: Metformin	Group 3: Glimepiride	Group 4: Healthy Control
Age (years)	43 ± 6	40 ± 3	41 ± 6	39 ± 8
Gender (Male/Female)	18/12	15/15	13/17	20/10
HbA1C(%)	6.9 ± 0.7	7.2 ± 0.8	7.9 ± 0.2	5.2 ± 0.3
Onset (months)	2 ± 0.5	12 ± 2	11 ± 4	--
Drug Dosage per day	--	Metformin-500 mg	Glimepiride-2 mg	--

**Table 2:** The primer sequences (forward and reverse) specific to the genes to assess the expression of BDNF and its interconnecting proteins encoding genes.

Gene	Orientation	Primer
APP	FORWARD	TGGCCAACATGATTAGTGAACC
	REVERSE	AAGATGGCATGAGAGCATCGT
GAPDH	FORWARD	GCCACATCGCTCAGACACC
	REVERSE	AATCCGTTGACTCCGACCTTC
AP2M1	FORWARD	TCTCGGCTTGTCCTAACACAG
	REVERSE	CTTACAGCCGCTCTCCTCTG
ESR1	FORWARD	CTCTTAGCTATGCCTGGGGC
	REVERSE	GGTGGATGTTTACTCCGCCA
BDNF	FORWARD	GATGCTGCAAACATGTCCATGAG
	REVERSE	TTTTGTCTGCCGCCGTTACC

## Data Availability

Data are contained within the article and supplementary materials.

## Supplementary Materials

The following supporting information can be downloaded at: https://www.mdpi.com/article/10.3390/biomedicines11112939/s1, Table S1: Molecular pathways associated with metformin and glimepiride. Figure S1: Gene ontology of overrepresented genes that predicted to be activated upon glimepiride treatment in T2DM. Figure S2: Gene ontology of overrepresented genes that predicted to be activated upon metformin treatment in T2DM.
