# Supplementary material for: Comparative Efficacy of Metformin and Glimepiride in Modulating Pharmacological Network to Increase BDNF Levels and Benefit Type 2 Diabetes-Related Cognitive Impairment
Source: Biomedicines. 2023 Oct 31;11(11):2939. doi: 10.3390/biomedicines11112939 (PMC10669717; doi:10.3390/biomedicines11112939)
Supplement: Supplementary file 1 [file biomedicines-11-02939-s001.zip › Table S1.pdf]

Supplementary Table S1: List of molecular pathways involved by metformin and glimipiride interactomes.

METFORMIN	
**S.No**	**Molecular Pathway**
1	mRNA Splicing - Minor Pathway
2	mRNA Splicing - Major Pathway
3	Generic Transcription Pathway
4	RIG-I/MDA5 mediated induction of IFN-alpha/beta pathways
5	Nucleotide-binding domain, leucine rich repeat containing receptor (NLR) signaling pathways
6	NOD1/2 Signaling Pathway
7	arginine degradation VI (arginase 2 pathway)
8	arginine degradation I (arginase pathway)
9	pyridoxal 5'-phosphate salvage pathway
10	EphrinA-EPHA pathway
11	Canonical NF-kappaB pathway
12	Insulin Pathway
13	C-MYC pathway
14	Nectin adhesion pathway
15	FGF signaling pathway
16	TRAIL signaling pathway
17	IGF1 pathway
18	EphrinB-EPHB pathway
19	BCR signaling pathway
20	Ras signaling in the CD4+ TCR pathway
21	Ceramide signaling pathway
22	IFN-gamma pathway
23	Noncanonical Wnt signaling pathway
24	PDGFR-beta signaling pathway
25	Canonical Wnt signaling pathway
26	HIF-2-alpha transcription factor network
27	p38 MAPK signaling pathway
28	EGF receptor (ErbB1) signaling pathway
29	Thrombin/protease-activated receptor (PAR) pathway
30	EPO signaling pathway
31	FAS (CD95) signaling pathway
32	mTOR signaling pathway
33	RAC1 signaling pathway
34	ALK1 pathway
35	Arf6 downstream pathway
36	JNK signaling in the CD4+ TCR pathway
37	S1P1 pathway
38	ATM pathway

39	TNF receptor signaling pathway
40	p53 pathway
41	ATR signaling pathway
42	PDGFR-alpha signaling pathway
43	Sphingosine 1-phosphate (S1P) pathway
44	Calcium signaling in the CD4+ TCR pathway
45	RhoA signaling pathway
46	Trk receptor signaling mediated by the MAPK pathway
**GLIMIPRIDE**	
1	Generic Transcription Pathway
2	Regulation of the Fanconi anemia pathway
3	Fanconi Anemia pathway
4	Nucleotide-binding domain, leucine rich repeat containing receptor (NLR) signaling pathways
5	NOD1/2 Signaling Pathway
6	Circadian rhythm pathway
7	Canonical NF-kappaB pathway
8	Glypican pathway
9	Arf1 pathway
10	Insulin Pathway
11	C-MYC pathway
12	Nectin adhesion pathway
13	FGF signaling pathway
14	TRAIL signaling pathway
15	IGF1 pathway
16	EphrinB-EPHB pathway
17	BCR signaling pathway
18	Ras signaling in the CD4+ TCR pathway
19	IFN-gamma pathway
20	Noncanonical Wnt signaling pathway
21	Regulation of p38-alpha and p38-beta
22	Alpha4 beta1 integrin signaling events
23	VEGFR1 specific signals
24	PDGFR-beta signaling pathway
25	p63 transcription factor network
26	Canonical Wnt signaling pathway
27	p38 MAPK signaling pathway
28	EGF receptor (ErbB1) signaling pathway
29	p75(NTR)-mediated signaling
30	Thrombin/protease-activated receptor (PAR) pathway
31	EPO signaling pathway
32	FAS (CD95) signaling pathway
33	mTOR signaling pathway
34	RAC1 signaling pathway
35	ALK1 pathway

36	Arf6 downstream pathway
37	JNK signaling in the CD4+ TCR pathway
38	S1P1 pathway
39	ATM pathway
40	TNF receptor signaling pathway
41	p53 pathway
42	ATR signaling pathway
43	PDGFR-alpha signaling pathway
44	Notch signaling pathway
45	Sphingosine 1-phosphate (S1P) pathway
46	Atypical NF-kappaB pathway
47	Calcium signaling in the CD4+ TCR pathway
48	RhoA signaling pathway
49	Trk receptor signaling mediated by the MAPK pathway
